# Quantifying Y chromosome loss in primary and metastatic prostate cancer by chromosome painting

**DOI:** 10.1371/journal.pone.0301989

**Published:** 2024-04-29

**Authors:** Sai Harisha Rajanala, Romina Ghale, Subhiksha Nandakumar, Kalyani Chadalavada, Gwo-Shu Mary Lee, Konrad H. Stopsack, Yu Chen, Gouri J. Nanjangud, Goutam Chakraborty, Philip W. Kantoff

**Affiliations:** 1 Department of Medicine, Memorial Sloan Kettering Cancer Center, New York, New York, United States of America; 2 Molecular Cytogenetics Core, Memorial Sloan Kettering Cancer Center, New York, New York, United States of America; 3 Department of Medical Oncology, Dana-Farber Cancer Institute, Boston, Masacheussets, United States of America; 4 Department of Urology, Icahn School of Medicine at Mount Sinai, New York, New York, United States of America; 5 Departments of Oncological Sciences Icahn School of Medicine at Mount Sinai, New York, New York, United States of America; 6 Tisch Cancer Institute; Icahn School of Medicine at Mount Sinai, New York, New York, United States of America; 7 Convergent Therapeutics, Inc., Cambridge, Masacheussets, United States of America; CNR, ITALY

## Abstract

Somatic Y chromosome loss in hematopoietic cells is associated with higher mortality in men. However, the status of the Y chromosome in cancer tissue is not fully known due to technical limitations, such as difficulties in labelling and sequencing DNA from the Y chromosome. We have developed a system to quantify Y chromosome gain or loss in patient-derived prostate cancer organoids. Using our system, we observed Y chromosome loss in 4 of the 13 (31%) patient-derived metastatic castration-resistant prostate cancer (mCRPC) organoids; interestingly, loss of Yq (long arm of the Y chromosome) was seen in 38% of patient-derived organoids. Additionally, potential associations were observed between mCRPC and Y chromosome nullisomy. The prevalence of Y chromosome loss was similar in primary and metastatic tissue, suggesting that Y chromosome loss is an early event in prostate cancer evolution and may not a result of drug resistance or organoid derivation. This study reports quantification of Y chromosome loss and gain in primary and metastatic prostate cancer tissue and lays the groundwork for further studies investigating the clinical relevance of Y chromosome loss or gain in mCRPC.

## Introduction

Y chromosome loss is the most common somatic genetic aberration in older men [1]. Loss of the Y chromosome found in hematopoietic cells is associated with increased risk of disease, including cancer, and mortality [2,3]. Our previous studies have demonstrated that loss of the Y chromosome gene *KDM5D* resulted in aggressive and docetaxel-resistant prostate cancer [4,5]. However, the impact of Y chromosome status, including loss or gain or changes in other structural features of the Y chromosome, in cancer tissue is still not fully understood due to a lack of relevant experimental models and tools that can be used to assess Y chromosome loss. Herein, we study complete and partial loss (loss of only Yq or Yp) of the Y chromosome in prostate cancer.

Investigating Y chromosome loss or gain is challenging mainly due to the difficulty in karyotyping patient tissue samples. The highly repetitive DNA sequences in the Y chromosome, coupled with low gene expression in adults, limit next-generation sequencing as a feasible method to analyze Y chromosome status in patient tissue samples [6]. We developed a novel fluorescence *in situ* hybridization (FISH) probe for chromosome painting that allowed us to visualize and quantify Y chromosome–related heterogeneity in prostate cancer cell lines and patient-derived organoids. Here, we present the results of our chromosome painting FISH probe system, which can be used to probe patient-derived organoids to gain an understanding of the X and Y chromosome status in prostate cancer.

## Results

### XY paint probe validation

**Fig 1:**
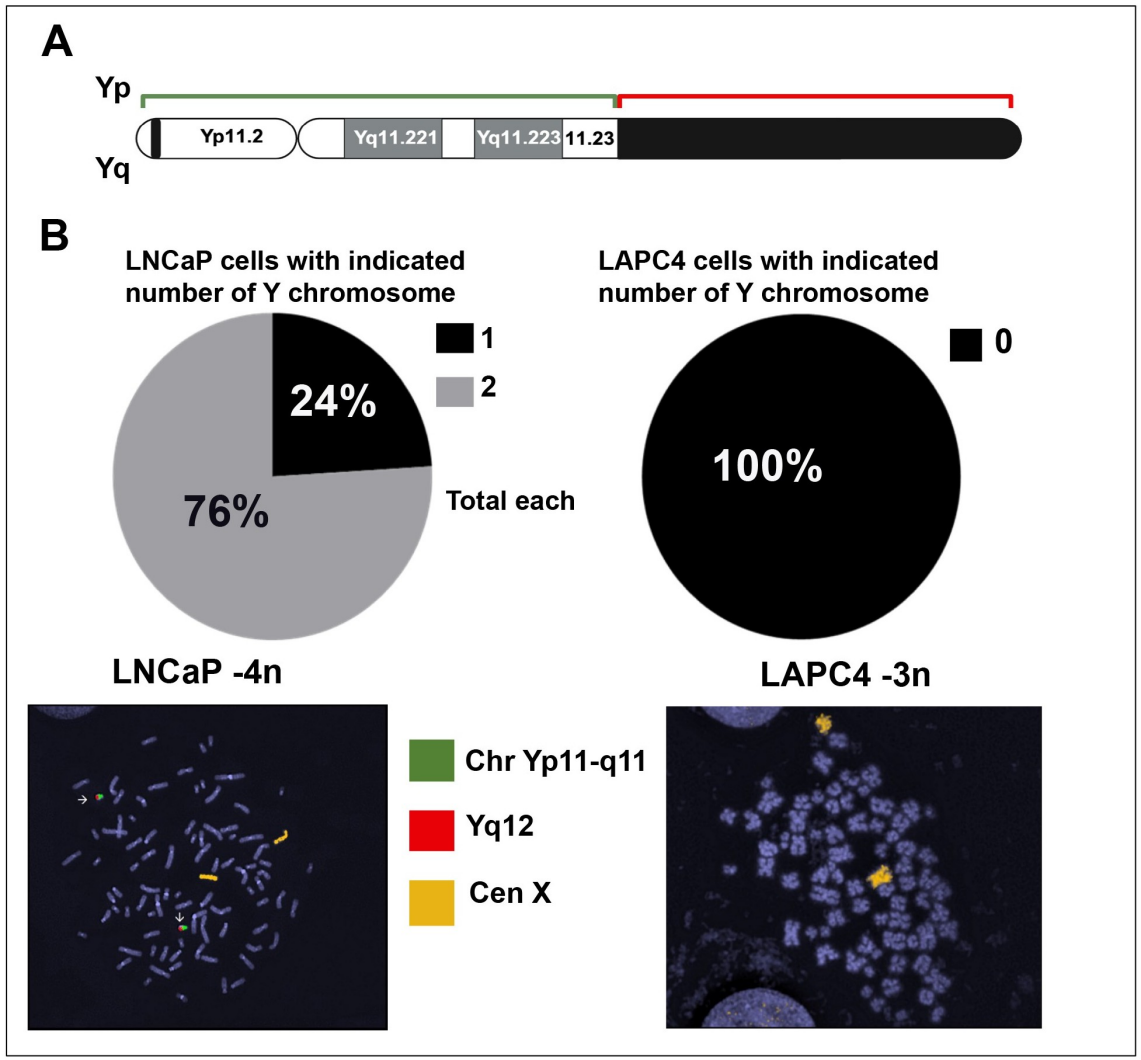
Y chromosome paint FISH probe design and validation. (A) FISH probe design. (B) XY paint probe validation in prostate cancer cell lines, LNCaP (left) and LAPC4 (negative control; right). Cen X, centromere X; FISH, fluorescence in situ hybridization.

Thirteen patient-derived metastatic prostate cancer organoid cultures were screened and tested using our FISH probe system, which marked the X chromosome as well as the euchromatic Yp (short arm) and heterochromatic Yq (long arm) regions on the Y chromosome with orange, green, and red fluorochromes, respectively (Fig 1A). We validated the XY paint probes in prostate cancer cell lines: LNCaP, which carries 2 copies of the Y chromosome, and LAPC4 (negative control), which has Y chromosome loss (Fig 1B) [7]. Using this system, we were able to identify prevalent Y chromosome loss in organoids.

### Y chromosome loss was detected in patient-derived prostate cancer organoids

**Fig 2:**
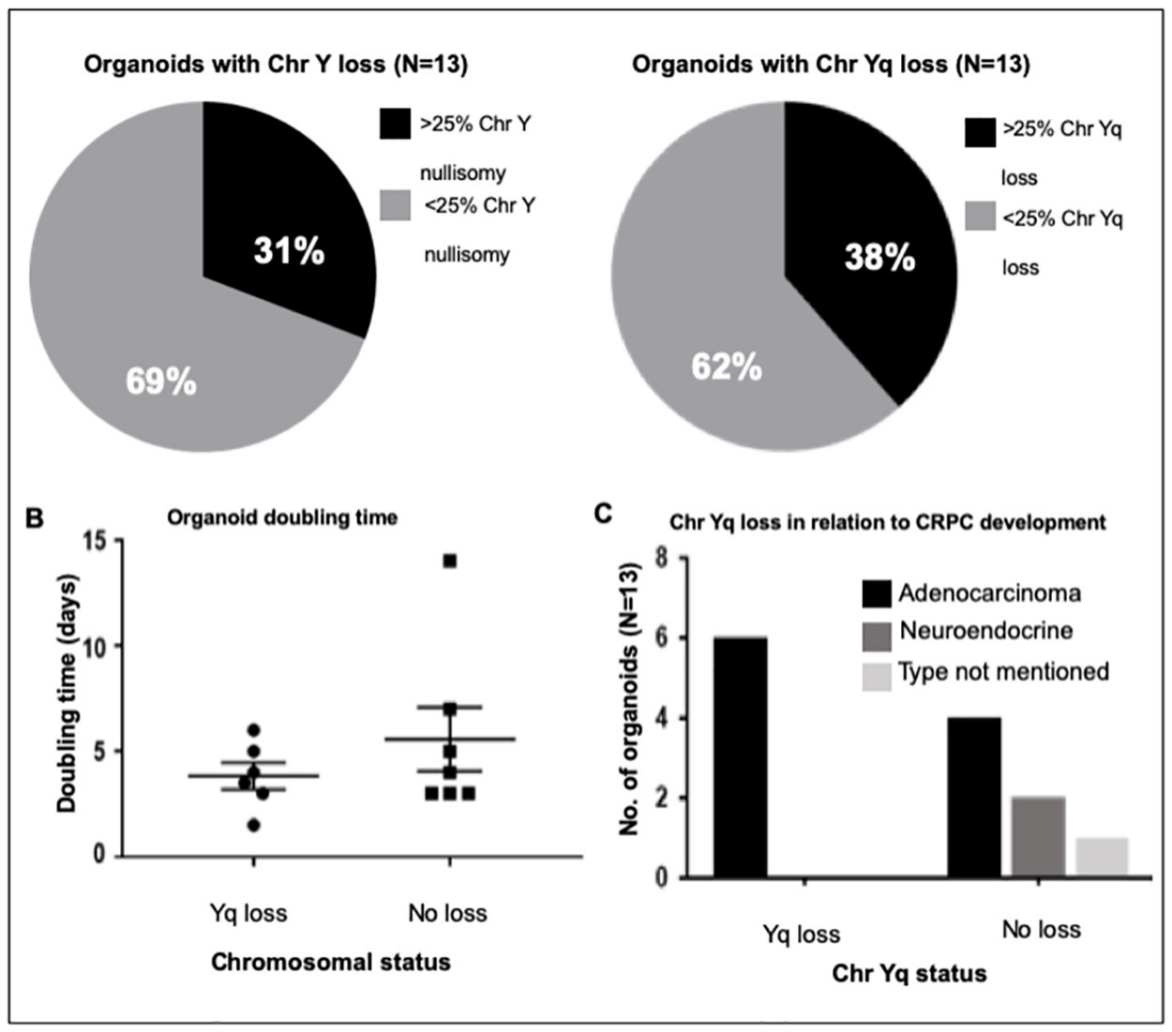
Characteristics of patient-derived prostate cancer organoids. (A) Percentage of organoids with loss of the chromosome Y (left) and Yq (right). (B) Doubling times of organoids. (C) Breakdown of organoids, stratified by type of prostate cancer tissue used for patient-derived organoid development and Y chromosome status. Chr, chromosome; CRPC, castration-resistant prostate cancer.

Six of 13 organoids (46%) were heterogenous in terms of Y chromosome status (Table 1), i.e. had sub-populations with variable Y chromosome number. Using our cutoff for classifying chromosomal loss (>40% chromosome Y or Yq loss), 31% (n = 4) of the organoids were classified with complete Y chromosome loss. Interestingly, 38% (n = 5) of the organoids were classified with Yq loss (partial loss of Y chromosome), which occurred more frequently than complete Y chromosome loss (Fig 2A).

**Table 1:** Characteristics of patient-derived metastatic prostate cancer organoids labelled with the XY paint FISH probes.

Name of organoids	Prior therapy*	Doubling time^ǂ^ (days)	Gleason grade	Complete Chr Y loss (%)	Yq loss (%)
MSKPCa 11	ADT, docetaxel	4	8 (4+4)	0	
MSKPCa 8	ADT, sipuleucel-T	3.5	8	44	44
MSKPCa 16	ADT	3	-	0	20
MSKPCa 14-matrigel	Degarelix	5	7 (4+3)	0	12
MSKPCa 17-matrigel	ADT (nilutamide and abiraterone), cabazitaxel	6	9 (4+5)	100	100
MSKPCa 9	ADT, sipuleucel-T	4	9 (5+4)	16	100
MSKPCa 10	ADT	3	8 (4+4)	8	4
MSKPCa 12	ADT	1.5	9 (4+5)	100	100
MSKPCa 15	ADT (enzalutamide and abiraterone)	5	7 (4+3)	44	100
MSKPCa 1	ADT, bicalutamide	3	9 (4+5)	0	0
MSKPCa 3	ADT, bicalutamide, docetaxel, carboplatin	7	-	0	0
MSKPCa 2	ADT, bicalutamide	3	-	0	4
MSKPCa 4	ADT, bicalutamide, docetaxel	14	7 (4+3)	0	0

*If clincal history specified the ADT regimen, those therapeutic interventions are specified in parenthesis.

^**ǂ**^Doubling time in days describes the organoid growth rate.

*ADT*, androgen deprivation therapy; *FISH*, fluorescence *in situ* hybridization; *Chr Y loss*, loss of chromosome Y; *Yq loss*, loss of Yq (long arm of the Y chromosome; defined as partial loss of Chr Y).

### Association between Y chromosome loss and prostate cancer features

Patient-derived prostate cancer organoids with loss of chromosome Yq had a mean growth rate of 3.8 days (median: 3.75, range: 1.5–6 days), compared to 5.6 days for organoids with no loss (median: 4, range: 4–14 days; *P* = 0.34) (Fig 2B). In our study and sample set, loss of the Yq chromosome was observed only when organoids were derived from patients with metastatic castration-resistant prostate cancer (mCRPC) (Fig 2C). These observations indicate that loss of the Yq chromosome could possibly be associated with a more aggressive form of prostate cancer.

### Y chromosome loss was observed in 36% of primary prostate cancer patient samples

**Fig 3:**
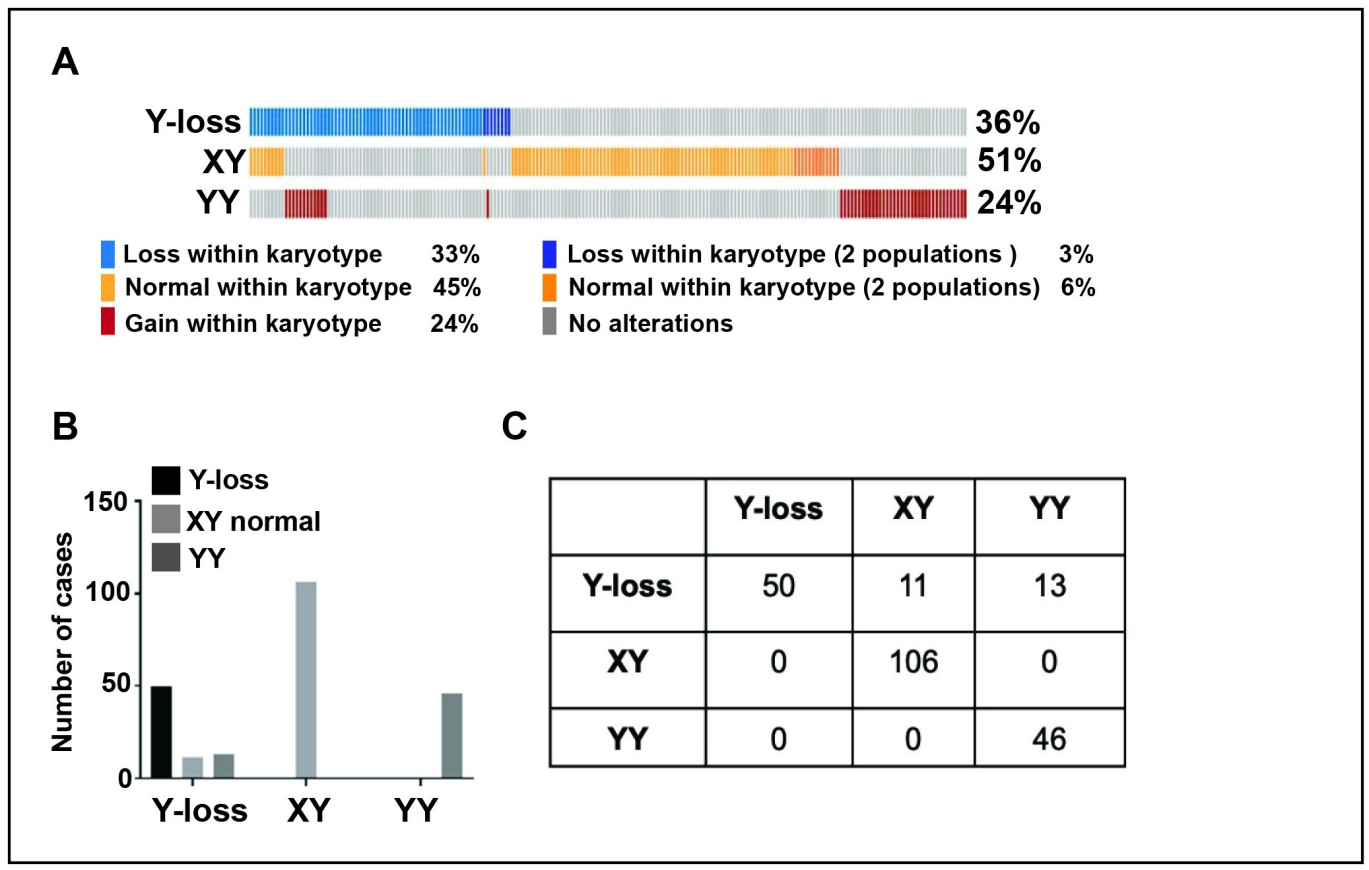
Characteristics of the Y chromosome in primary prostate cancer samples from patients using Mitelman’s Database. (A) Y chromosome status in primary prostate cancer samples. (B) Y chromosome heterogeneity in primary prostate adenocarcinoma samples. Chi-square test reported significance.

The Mitelman Database of Chromosome Aberrations and Gene Fusions in Cancer, a collection of data mined from the literature, also was queried to analyze the status of Y chromosome loss in primary prostate cancer patient samples [8]. Interestingly, Y chromosome loss occurred in 36% (74 of 203 total cases) of primary prostate cancer samples (Fig 3A). Within this dataset, there were also primary tissue samples containing mixed populations of cells, some of which expressed loss and some expressed gain of the Y chromosome. Interestingly, this heterogeneity in terms of Y chromosome status was observed only in samples with Y chromosome loss. This observation raises the question that Y chromosome loss could be a measurable indicator of chromosomal instability in prostate cancer (chi square test p<0.0001) (Fig 3B).

## Discussion

This is, to our knowledge, the first study to analyze Y chromosome status in both primary prostate cancer tissue (using data from the Mitelman Database of Chromosome Aberrations and Gene Fusions in Cancer) and patient-derived metastatic prostate cancer organoids. Importantly, we were able to specifically identify prevalent Y chromosome loss in patient-derived prostate cancer organoids using our chromosome painting FISH probe system. This work provides novel insights into the potential role of changes in Y chromosome status in disease progression in patients with prostate cancer. Using patient-derived metastatic prostate cancer organoids in lieu of metastatic prostate cancer patient samples, we found that primary and metastatic prostate cancer samples have similar prevalence of Y chromosome loss. Organoids derived from patient samples are efficient avatars for tumor samples, as they allow functional characterization using assays such as doubling time or drug sensitivity (for future studies). This observation further strengthens our hypothesis that Y chromosome loss is a core feature of prostate cancer and not a consequence of metastasis or culturing of organoids. Loss of the Y chromosome was observed at a comparatively higher frequency in primary tissue samples comprised of mixed populations expressing chromosomes XY or gain of the Y chromosome than in ones with no loss. These observations collectively present the need to further investigate Y chromosome loss as a marker of overall chromosomal instability in prostate cancer.

## Materials and methods

### Organoid culture

Patient-derived prostate cancer organoids were obtained from the Yu Chen Lab at Memorial Sloan Kettering Cancer Center (MSK). All organoids were derived from metastatic human prostate cancer tissue and cultured using conditions previously outlined [9]. We classified all organoid samples with >40% chromosome Y or Yq loss as having chromosome Y or Yq loss, respectively; 40% was defined as the cutoff as it was close to the mean (~38%) of Yq12 loss in the organoid set. Mixed populations are defined as organoid cultures that had subpopulations based on chromosome Y status, e.g., samples with 44% Y loss, 76% no loss (XY).

This study as well as the use of prostate cancer cell lines and patient-derived organoids described herein were approved by MSK’s institutional review board. Informed consent (written) for tissue collection and generation, storage and use of the organoids was obtained from all patients at MSK.

### FISH probe design and analysis

FISH analysis was performed on cell lines harvested and fixed in methanol and acetic acid at a 3:1 ratio, respectively. A 3-color probe was designed to detect loss and structural alterations of the Y chromosome, and the X chromosome served as the control. The probe consisted of plasmid mapping to heterochromatic region Yq12 (clone Y84; labelled with spectrum red), whole chromosome paint for euchromatic region Yp11-q11 (labelled with spectrum green), and whole chromosome paint for the X chromosome (labelled with spectrum orange). Probe labelling, hybridization, post-hybridization washing, and fluorescence detection were performed according to procedures established at the Molecular Cytogenetics Core Facility at MSK. Microscopy slides mounted with organoid samples were scanned using a Zeiss Axioplan 2 imaging epifluorescence microscope (Carl Zeiss Microscopy, LLC, White Plains, NY), which was equipped with Isis imaging software (MetaSystems Group, Inc, Medford, MA). Images were acquired by scanning the entire hybridized area using 63X objective lens to assess quality of hybridization and signal pattern. Following the initial scan, for each cell line, a minimum of 25 metaphase chromosome spreads were imaged and analyzed. Chromosomes were counted to infer ploidy, and the call for chromsome loss or gain was in relation to ploidy.

### Mitelman database query

We used the ISCN-SNAKE tool (https://gitlab.com/VizeacoumarLab/ISCN-SNAKE) (Download date Jan 20, 2021) [10] to parse data from the Mitelman Database of Chromosome Aberrations and Gene Fusions in Cancer [https://mitelmandatabase.isb-cgc.org/search_menu] [8]. We specifically queried the database by filtering cases indicated as ‘prostate’ for topography, and cases with this topography subsequently were filtered for morphology indicated as only ‘adenocarcinoma’ (query: “Topography:Prostate//Morphology:Adenocarcinoma").

## Supporting information

S1 Fig Prostate cancer organoid demonstrating no Y loss XY paint of organoids demonstrating no Y loss. (TIF)

S2 Fig Prostate cancer organoid demonstrating partial Y loss XY paint of organoid demonstrating Y loss in some cells and no Y loss in others. (TIF)

S3 Fig Prostate cancer organoid demonstrating Y loss XY paint of organoid demonstrating complete Y loss. (TIF)
