# Supplementary figures and images for: Quantifying Y chromosome loss in primary and metastatic prostate cancer by chromosome painting
Source: PLoS One. 2024 Apr 29;19(4):e0301989. doi: 10.1371/journal.pone.0301989 (PMC11057730; doi:10.1371/journal.pone.0301989)

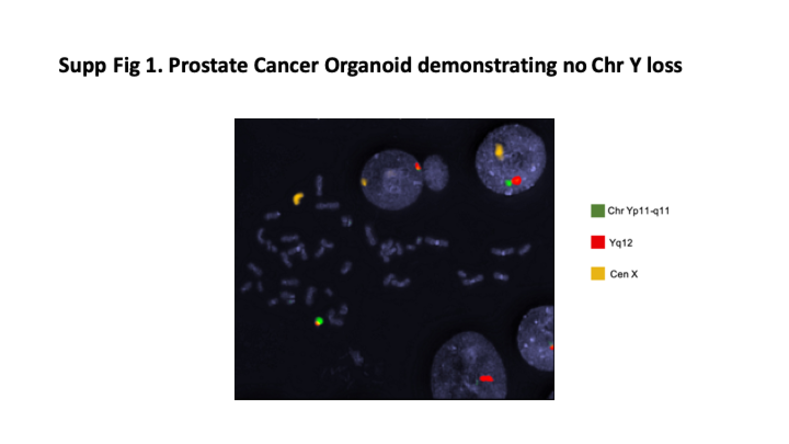

Supplement: S1 Fig — (TIF) [file pone.0301989.s001.tif]

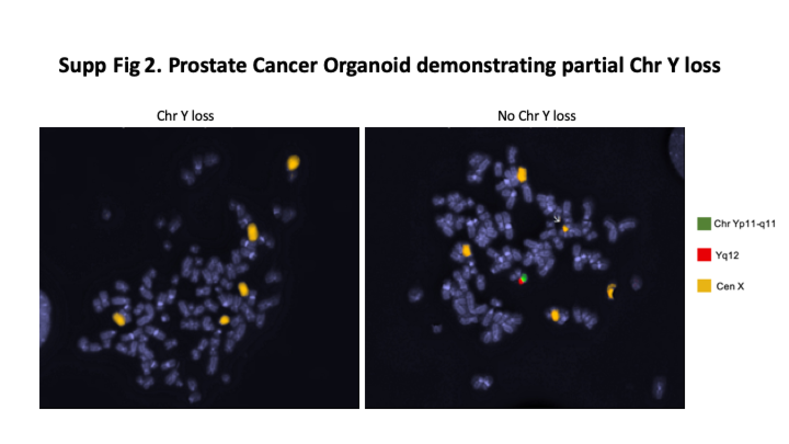

Supplement: S2 Fig — (TIF) [file pone.0301989.s002.tif]

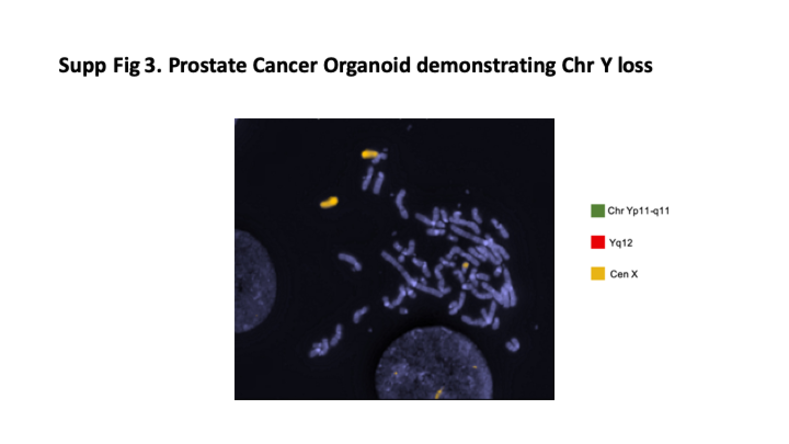

Supplement: S3 Fig — (TIF) [file pone.0301989.s003.tif]
